# Renewable, Degradable, and Recyclable Polymer Composites

**DOI:** 10.3390/polym15071769

**Published:** 2023-04-02

**Authors:** Aleksander Hejna

**Affiliations:** Institute of Materials Technology, Poznan University of Technology, Piotrowo 3, 60-965 Poznań, Poland; aleksander.hejna@put.poznan.pl

The substantial plastic pollution reaching almost every area of our planet and every aspect of human lives is pushing the polymer sector towards a circular economy, which would significantly limit its environmental impact. Such a narrative is driven by changing human perceptions, prompting trends towards sustainable development, which have transformed into various local, national, international, or even global regulations such as the Kyoto Protocol. The Kyoto Protocol aims to reduce greenhouse gases emissions often associated with the use of fossil fuels, including carbon dioxide, in the manufacturing of petroleum-based polymers, among others [1]. In recent years, the European Union (EU), through its directives and climate targets, has required its member states to orient their industries towards renewable raw materials. In 2019, the European Union presented the European Green Deal as a roadmap for obtaining a sustainable EU economy [2]. As a part of this, additional guidelines have been developed. In 2020, the EU presented “A new circular economy action plan for a cleaner and more competitive Europe” [3], as well as the 2030 Climate Target Plan [4]. One of the main goals of the European Green Deal is the reduction in greenhouse gases emissions (by at least 55% by 2030 compared to 1990).

The polymer sector is one of the most critical for realizing such a transformation, since plastics are commonly used in medicine, food preservation, energy production and storage, water purification, transport, construction and building, electronics and the military sector [5,6,7,8]. These applications are often crucial for a functioning society, and banning the use of plastics would set back the development of these sectors by decades. Therefore, to maintain the standard of life of the global population and simultaneously save the Earth, it is the responsibility of researchers and industry to seek more environmentally friendly solutions, including renewable, degradable and recyclable materials. The development of such solutions would fit into the circular economy approach. Currently, the application of renewable, degradable and recyclable polymers covers not only the traditional 3Rs (Reduce, Reuse and Recycle), but also strongly takes inspiration from the new 9Rs (Refuse, Rethink, Reduce, Reuse, Repair, Refurbish, Remanufacture, Repurpose, Recycle and Recover) [9]. The traditional 3Rs are well-known and clear, but the new—and most critical—aspects, “Refuse, Rethink and Reduce”, encourage new ways of thinking. These aspects are associated with innovations in plastic production, including predictions of product lifetimes, engineering their degradation or providing novel functionalities, which pushes this sector towards circular economy, which is schematically presented in Figure 1.

However, a circular economy in the plastics sector is still an unattainable dream, which implicates the need for novel solutions in the field of renewable, degradable and recyclable polymer composites. In line with the ever-increasing demands for such innovations, this Special Issue, entitled “Renewable, Degradable, and Recyclable Polymer Composites” has been proposed by the *Polymers* journal from the MDPI family. The potential topics covered by this Special Issue are (but are not limited to) “recycling of polymer materials as matrices for composites”; “waste-based or recycled fillers or additives for polymer materials”; “novel resources for renewable polymer composites”; “biodegradability and composting of polymer composites”; “environmental impacts of sustainable polymer composites”; and “life cycle assessment of waste-based polymer composites”. Contributions from academia and industry are strongly encouraged, as a purely holistic approach including different points of view would lead to a rapid transition towards a circular economy in the plastics sector.

## Figures and Tables

**Figure 1:**
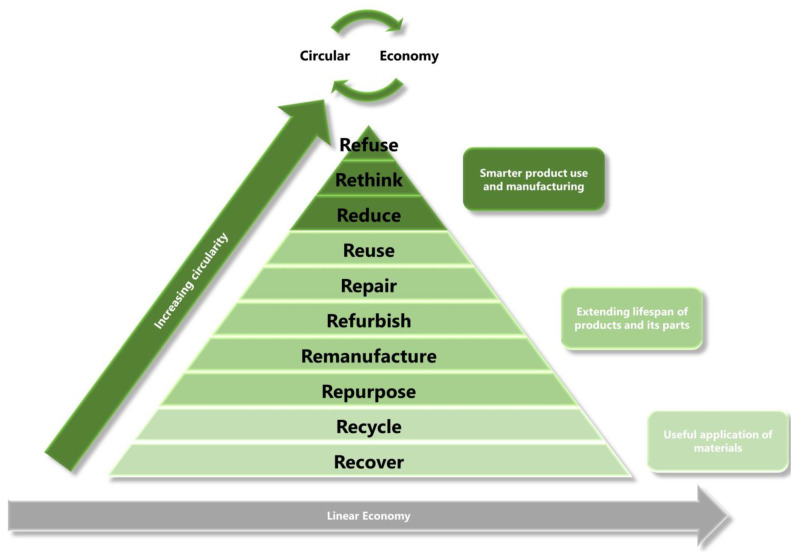
A general view of the desired transition from a linear economy to a circular economy.
